# Effects of Moderate Exercise on Relieving Mental Load of Elementary School Teachers

**DOI:** 10.1155/2015/192680

**Published:** 2015-03-10

**Authors:** Shing-Hong Liu, Da-Chuan Cheng, Jia-Jung Wang, Tzu-Hsin Lin, Kang-Ming Chang

**Affiliations:** ^1^Department of Computer Science and Information Engineering, Chaoyang University of Technology, Taichung 41349, Taiwan; ^2^Department of Biomedical Imaging and Radiological Science, China Medical University, Taichung 40402, Taiwan; ^3^Department of Biomedical Engineering, I-Shou University, Kaohsiung 84001, Taiwan; ^4^Department of Cardiology, Lin-Shin Hospital, Taichung 40867, Taiwan; ^5^Department of Photonics and Communication Engineering, Asia University, Taichung 41354, Taiwan; ^6^Department of Medical Research, China Medical University Hospital, China Medical University, Taichung 40402, Taiwan

## Abstract

Long-term endurance exercise could increase activity of parasympathetic nervous and decrease activity of sympathetic nervous at rest. However, previous studies all focused on the effect of endurance training on heart rate variability (HRV) for athletes or sedentary subjects. In Taiwan, elementary school teachers teaching and processing the children's and administrative problems always stand and walk. They will sit down only when they review and correct the students' home work. Thus, the goal of this study was to elucidate the beneficial effect of moderate intensity exercise on relieving mental load of elementary school teachers. There were 20 participants in the exercise group and another 20 participants in the nonexercise group. The exercising teachers performed 12 weeks of moderate intensity exercise training for an average of 30 minutes per day, 3 times per week. HRV was measured before and after the 4th, 6th, and 12th weeks. The time and frequency domain parameters of HRV all had significant increases between the beginning and after 12 weeks of training. However, the time and frequency domain parameters of HRV in the nonexercise group had significant decreases between the beginning and after 12 weeks of training. The long-term moderate exercises can relieve mental load of elementary school teachers. Moreover, age was the considerable factor affecting HRV in this study.

## 1. Introduction

When people feel physical or psychological stress, the sympathetic nervous system (SNS) becomes more active. When this stressor disappears, the parasympathetic nervous system (PNS) reduces the heart rate and breathing rate. The response of the autonomic nervous system can be monitored using the heart rate variability (HRV), which is derived from the heartbeat interval time series [1, 2]. Some studies have shown that physical tasks influence the HRV [3–5]. Psychological depressive and anxiety disorders also affect the HRV [6–8]. Jorna introduced the background information on concepts of mental load and stress and the possibility of indexing them by means of spectral analysis of heart rate (HR) [9]. Garde et al. studied effects of mental and physical demands on HRV during computer work [10]. Therefore, HRV is an established noninvasive tool that can be used to explore the degree of mental load during the work.

During exercise, HR increases linearly with the exercising intensity. The training status influences the response of the heart rate, because the SNS and PNS interact with each other [1]. Thus, there have been many studies that have used the HRV to explore the response of the autonomic nervous system to the effects of sports, at rest or during exercise. The results of some studies on the effects of endurance exercise showed resting bradycardia and no significant difference in HRV [11–13]. However, a number of studies have shown different results in which the RR interval and high-frequency spectral power of HRV all had significant increases in endurance exercise training [14, 15]. HRV was also used to evaluate daily intensity training to improve the effects of cardiac and respiratory fitness [16–20]. The results of these studies showed that the HRV could be used as a guide for intensity training individually.

Mental load as a concept serves as an intermediate between imposed and perceived demands. It also is viewed as an index of discrepancy between required capacities of the information processing system and available capacities at any given time [5, 9]. Moreover, if people on a high mental load work for a long period, they will be fatigued and stressful enough to be posed to serious health problems, such as hypertension and cardiac failure [21, 22]. A number of studies examined the effects of aromatherapy and music therapy for relieving mental load [23–26]. The results all showed that there were significant effects with these therapies. Raglin discussed the issue of exercise and mental health. He thought that exercise could result in either beneficial or detrimental changes in mental health to be dependent on the dosage employed [27]. Bitonte and DeSanto II studied the effects of mandatory exercise on the prevention of the mental illness [28]. We did not find any research to discuss the benefits of the sport therapy for relieving the mental load in element school teachers.

In Taiwan, elementary school teachers must provide more time to process children's problems beyond teaching every day. Teachers also have various administrative duties. When teachers are teaching and processing the children's and administrative problems, they always stand and walk. They will sit down only when they review and correct the students' home work. Therefore, all elementary school teachers are at risk of suffering from work-related stress. Moreover, elementary schools in Taiwan have summer and winter vacations. Within these vacations, teachers do not do anything and rest at home. Thus, they feel fully relaxed before school begins. Thus, the goal of our study was to clarify the beneficial effects of moderate intensity exercise on alleviating mental load of elementary school teachers within the semester. In our study, 20 teachers voluntarily participated in twelve weeks of moderate intensity exercise in the last three weeks after beginning a school session. Another 20 teachers were assigned to a nonexercise group that did not do any regular exercises within the same time. We compared physiological parameters such as weight, body fat, and HRV between the two groups at different months. Since age could affect the effects of the exercise training [4, 29], we also considered it as a variable in this study.

## 2. Materials and Methods

### 2.1. Participants

This experiment was approved by the Asia University Medical Research Ethics Committee. We recruited 40 elementary school teachers who all gave consent to attend this study and did not have hypertension or heart conditions, because a number of studies have shown that heart conditions such as arrhythmia, myocardial ischemia, and a history of heart failure affect the HRV [30, 31]. The Beck Anxiety Inventory (BAI) was used to estimate the anxiety degree of every participant. The participant was excluded from this study whose score was above 10. Moreover, participants all do not ride the bike or run to school and have the habits of exercise every day. They all have a healthier lifestyle. Because the duration of exercise training would be about three months, we hoped all participants to have the interest to participate this study. Thus, the voluntary participants self-selected based on their interest for the moderate intensity exercise training or nonexercise.

### 2.2. Outcome Measures

For the heart rate measurements and HRV analysis, we employed a handheld HRV meter (type LR8Z11, made by Yunyin Co., Ltd., Taiwan). It measures one lead electrocardiogram (ECG) with a sampling rate of 500 Hz and resolution of 12 bits. Blood pressure was measured using an electronic blood pressure monitor (type HEM-7210, manufactured by OMRON Co. Ltd., Japan). Body fat and weight were measured by a device (type 01-TT-BF800, manufactured by TANITA Co. Ltd., Japan). The software of the handheld HRV meter could analyze the time and frequency domain parameters of HRV.

Using a discrete Fourier transform, the HRV is derived from the heartbeat interval time series, for which the resampling rate is 4 Hz. Low-frequency power (LF: 0.04–0.15 Hz), high-frequency power (HF: 0.15–0.4 Hz), and the total power (TP: 0.04–0.4 Hz) were calculated. LF is affected by the vagal and SNS, and HF is affected by the PNS [2]. The time domain parameters are the simplest variables to describe the response of HRV that includes the standard deviation of the normal-normal interval (SDNN), the square root of the mean of the sum of the squares of differences between adjacent normal-normal intervals (RMSSD), and the standard deviation of differences between adjacent normal-normal intervals (SDSD) [2]. The time and frequency domain parameters are strongly correlated with each other. SDNN is correlated with TP and RMSSD and SDSD are correlated with HF [2].

### 2.3. Experiment Procedure

Moderate intensity exercise is defined here as walking 3 kilometers at about 6 km/hour speed. The participants of the exercise group did 12 weeks of training three times a week at the school's playground when students had all left school. If weather was raining, they would walk in the corridor of the school's building. Each participant provided personal information including gender, age, years of employment, height, and relationship status. Before the exercise course, participants did an initial measurement including weight, body fat, blood pressure, and HRV as the baseline. Then, these physiological parameters were observed again after the 4th, 8th, and 12th weeks of training.

Because teachers cannot smoke in the school according to the government laws, we did not consider the smoking problem. But we asked participants not to drink the caffeine beverage before measuring HRV by at least 4 hours. The measurements of HRV were conducted in a bright and quiet room, and the ambient temperature was kept at 22–25°C. Participants were encouraged to relax in a sitting position in a comfortable chair with arm supports, which provided easy access to attach the electrodes. They were asked to rest for 5 to 10 minutes before HRV recording, which was done for 5 minutes. Within the recording time, participants controlled their respiration rate at a fixed rate of 12 breaths per minute.

### 2.4. Statistics

We employed the SPSS 12.0 software package to conduct paired *t*-test for the intergroup differences, one-way ANOVA for treatment over time, and two-way ANOVA for comparison of each treatment over time. Significance for the *P* value was set at 0.05. Descriptive statistics were represented as the mean ± standard deviation. TP, LF, HF, SDNN, SDSD, RMSSD, and the R-R interval time (RRI) of ECG were used to indicate the response of the autonomic nervous system.

## 3. Results

Detailed participant information is shown in Table 1. There were 3 male participants and 17 female participants in the exercise group (average age: 43.8 ± 4.5 years), while there were 6 male participants and 14 female participants in the nonexercise group (average age: 43.0 ± 4.9 years). The middle age subgroup (>40 years) had 14 in the exercise group and 15 teachers in the nonexercise group. In the two groups, only BMI had a significant difference.

The baseline results and those obtained at the 4th, 8th, and 12th weeks of exercise training are shown in Table 2. We compared the changes of these physiological parameters between baseline and after different weeks of training. TP, HF, RMSSD, and SDSD were found to be increased and had significant differences after 4 weeks of training. RRI also had increased, but it did not have a significant difference. After 8 weeks of training, TP, LF, HF, RRI, SDNN, RMSSD, and SDSD had also increased, but only RRI had a significant difference. After 12 weeks of training, TP, LF, HF, RRI, SDNN, RMSSD, and SDSD not only were increased but also had very significant differences. However, the weight and body fat did not have any changes at the 4th, 8th, and 12th weeks.

Table 2 also shows the results of the nonexercise group. TP, LF, HF, SDNN, RMSSD, and SDSD were decreased at different weeks and all had significant differences after 12 weeks. The RRI was altered over the duration of this study. The weight and body fat also did not have any changes at the 4th, 8th, and 12th weeks. We compared the time and frequency domain parameters of subjects' HRV between the two groups over time. TP, LF, HF, RRI, SDNN, RMSSD, and SDSD had significant differences.

We compared the results of the exercise group and the non-exercise group at different weeks in Table 2. In the beginning, the weight, body fat, and HRV parameters did not have any significant differences. After 4 weeks, TP, HF, RRI, SDNN, RMSSD, and SDSD all had significant differences. Moreover, there was a very significant difference after 12 weeks. The weight and body fat did not have any significant differences at the 4th, 8th, and 12th weeks.

After controlling for the age variable, we conducted an analysis of the subgroup regarding the performance of the moderate intensity exercise.  Table 3 shows the results of the young subgroups at the different weeks. TP, HF, RRI, SDNN, RMSSD, and SDSD all had a small increase, and only RRI had significant differences at the 4th and 12th weeks.  Table 3 shows the results of the middle age subgroup at the different weeks. TP, LF, HF, RRI, SDNN, RMSSD, and SDSD all had substantial increases. But only TP, HF, RMSSD, and SDSD had significant differences at the 4th and 12th weeks but not RRI. We compared the time and frequency domain parameters of subjects' HRV between the young and middle age groups over time. No parameters of HRV had significant differences between two subgroups.

## 4. Discussions

The effects of endurance exercise training have been studied for many years [12, 14–16]. The goals of previous studies were to accurately quantify how an individual responds to training or overtraining. Moreover, they assessed whether the training responses were optimal in terms of producing advantageous physiological adaptations and improving performance. They have shown that the resting HR decreases slightly after endurance training for sedentary subjects or athletes [15, 32]. This phenomenon was due to a decrease in the intrinsic rhythmicity of the heart and an increase in the predominance of PNS control [16]. Some cross-sectional studies using HRV have found that trained individuals increased TP, HF, LF, and indices in the time domain compared to their untrained counterparts [32–34].

The work of the elementary school teacher is very different to normally sedentary individuals who work in the office because teachers always stand and walk when they are teaching and processing the children's and administrative problems. They sit down only when they review and correct the students' home work. Moreover, they have two long vacations (two months of summer vacation and one month of winter vacation) in one year to fully relax. Melanson and Freedson studied the resting heart rate of normally sedentary individuals after 16 weeks of training [32]. The time and frequency domain parameters of subjects' HRV remained unchanged in nonexercising controls. But, an exercise program of moderate to vigorous intensity produces increases in the time and frequency domain parameters of HRV for PNS control after 12 weeks. However, in our study, Table 2 shows that the time and frequency domain parameters of subjects' HRV and the resting RRI in the exercise group also have significant increases over time. According to the guidelines for HRV [2], these results represent the autonomic nervous system enhancing the activity of PSN control. In the nonexercising group, the time and frequency domain parameters of subjects' HRV were all decreased with increased working days. Moreover, these parameters had the significant differences between the beginning and after 12 weeks of training. Thus, we could find that the activity of their PSN was reducing within 12 weeks. Jorna presented that the responses of HRV were to increase the activity of SNS control and restrain the PNS control when people were on a high mental load [9]. Thus, these results maybe describe one problem. Teachers could fully relax within two long vacations. They are very busy within the semester. Thus, their mental load would easily be affected by their work. This phenomenon hardly happens with normally sedentary individuals. But if teachers do the moderate exercise three times a week, they can relax their mental load within the semester.

Although, we did not use the BAI to test the nonexercise group after 12 weeks, the results in the nonexercise group showed that their PNS control had been restrained. Thus, teachers need some methods to relieve their mental load within the semester to avoid the psychological depressive or anxiety disorders. Chang and Shen [23] and Liu et al. [24] proposed that the aromatherapy treatment could relieve the mental load of elementary school teachers. But the study of Chien et al. has shown that the aromatherapy treatment may have a persistent short-term effect on HRV with an increase in PNS modulation [35]. The results of this study show that the moderate intensity exercise training may have a persistent long-term effect to enhance the activity of PNS control for the elementary school teachers. This result is similar to those by Melanson and Freedson [32], Tulppo et al. [34], and Buchheit et al. [36].

Zhang demonstrated that age had a greater impact on HRV than gender. The older age group had a consistently lower LF and HF compared with the younger group [37]. He defined the young group (age < 30 years) and older group (age > 50 years). However, in Taiwan, the retired age of elementary school teacher is 50 years. Therefore, we hardly find the elder teachers (age > 50 years) to attend this study. We used 40 years to separate the young and middle age subgroup in the exercise group. Results of this study in the PNS modulation are very similar to the results of aromatherapy treatment by Liu et al. [24]. The HRV in the young subgroup did not have the effect after the moderate intensity exercise training but HRV in the middle age group had the significant effect. However, because the sample of the young group is much smaller than that of the middle age group, the results may have some tolerance.

In Table 2, the changes of time and frequency domain parameters of HRV at the 8th week are very different from results of the other weeks. The reason could be that the participating teachers were appraised for their abilities and performance to teach students by governmental staff at the 8th week. Teachers had to prepare many documents and practice teaching skills before this week. Thus, teachers all felt very anxious about the appraisal of their work. Therefore, the time and frequency domain parameters of HRV all had increased, but there were no significant differences.

We had expected that the weight and body fat of participants would decrease after the exercise training. But the two parameters did not have any change during this training trail. In this study, we had assigned one elementary school teacher to supervise the exercise program. Moreover, we did not use the exercise heart rate to control the training stimulus for all subjects. Thus, the intensity of exercise training maybe be not enough to change the physical states of subjects during this trial. However, the purpose of this study focuses on the effect of moderate exercise on relieving the mental load. We did not emphasize what exercise strength is better. But, the results of this study showed that the moderate exercise for a long-term training could relieve the mental load. This result was very similar to the suggestions of Bitonte and DeSanto II that thirty minutes a day three to five times a week of exercise for medical students had the benefits of mental illness prevention and health improvement [28].

## 5. Conclusion

We conducted this trial to examine the effect of moderate exercise for a long-term training on relieving mental load of elementary school teachers. The response of the automatic nervous system had a significant change to enhance the activity of PNS control after the 12 weeks of training. However, we found that the automatic nervous system in the nonexercise group also had a significant change to restrain the activity of PNS control after 12 weeks, because work stress of teachers may be accumulated over the time. Moreover, the middle age teachers found it easier to enhance their activity of PNS than the young teachers for a long-term training. But the age variable did not affect the time and frequency parameters of HRV over time.

## Figures and Tables

**Table 1:** Participant information.

Items	Control group *N* = 20	Experiment group *N* = 20	*P* value
Gender	Male: 6 Female: 14	Male: 3 Female: 17	

Age (years)	43.0 ± 4.9 Elder (>40): 15 Young (≤40): 5	43.8 ± 4.5 Elder (>40): 14 Young (≤40): 6	0.595

Weight (Kg)	60.5 ± 10.9	54.7 ± 7.2	0.055

Body fat (%)	29.1 ± 6.2	27.2 ± 6.1	0.33

BMI (Kg/m^2^)	23.3 ± 3.5	21.3 ± 2.1	0.019^*^

Mean ± SD; ^*^ *P* < 0.05.

**Table 2:** HRV ANOVA results for exercise and time effects and the interaction of both effects.

Parameters	Baseline		4th week		8th week		12th week		Interaction
	EG (*n* = 20)	CG (*n* = 20)	EG (*n* = 20)	CG (*n* = 20)	EG (*n* = 20)	CG (*n* = 20)	EG (*n* = 20)	CG (*n* = 20)	
TP (ms^2^)	10.36 ± 1.01	10. 4 ± 1.1	10.72 ± 0.9^*^	10.2 ± 0.9^‡^	10.6 ± 0.8	10.2 ± 0.8^‡^	11.2 ± 0.8^***^	9.8 ± 0.8^∗∗∗§^	0.002^**^
LF (ms^2^)	9.62 ± 1.2	9.7 ± 1.1	9.82 ± 1.2	9.54 ± 1.1	9.9 ± 1.0	9.6 ± 0.9	10.5 ± 1.0^***^	9.2 ± 1.0^∗∗§^	0.002^**^
HF (ms^2^)	9.46 ± 1.1	9.5 ± 1.2	10.02 ± 1.0^**^	9.2 ± 1.1^§^	9.8 ± 0.9	9.2 ± 1.0^§^	10.3 ± 0.9^***^	8.8 ± 1.0^∗∗∗§^	0.011^*^
RRI (ms)	715.4 ± 196.8	689.1 ± 139.5	775.8 ± 168.4	718.0 ± 137.9^†^	787.3 ± 172.6^*^	746.4 ± 81.5^*^	811.4 ± 140.7^**^	709.1 ± 130.3^§^	0.686
SDNN (ms)	40.2 ± 18.5	37.4 ± 15.4	48.5 ± 26.5	34.2 ± 13.4^§^	45.1 ± 24.5	33.4 ± 11.7^‡^	59.3 ± 30.3^***^	29.4 ± 10.4^∗∗∗§^	0.008^**^
RMSSD (ms)	32.6 ± 16.1	32.5 ± 18.9	42.8 ± 21.9^**^	27.4 ± 13.7^∗§^	36.4 ± 18.4	26.5 ± 12.8^∗§^	46.4 ± 22.8^***^	22.2 ± 10.5^∗∗∗§^	0.013^*^
SDSD (ms)	33.0 ± 16.3	33.0 ± 16.3	43.3 ± 22.2^**^	27.69 ± 13.8^∗§^	36.9 ± 18.6	26.77 ± 13.0^∗§^	47.0 ± 23.2^***^	22.44 ± 10.6^∗∗∗§^	0.012^*^
Weight (kg)	54.7 ± 7.2	60.5 ± 11.0	54.6 ± 7.0	60.64 ± 11.09	54.9 ± 7.2	60.67 ± 11.08	54.9 ± 6.9	60.49 ± 10.99	1.000
Body Fat (%)	27.2 ± 6.1	29.1 ± 6.2	26.5 ± 6.1	29.0 ± 6.7	27.2 ± 5.8	30.9 ± 11.1	27.3 ± 6.2	29.2 ± 6.8	0.927

Mean ± SD; CG: nonexercise group; EG: exercise group; TP: total power; LF: low-frequency power; HF: high-frequency power; RRI: R-R interval; SDNN: standard deviation of the normal-normal interval; RMSSD: square root of the mean of the sum of the squares of differences between adjacent normal-normal intervals; SDSD: standard deviation of differences between adjacent normal-normal intervals; ^*^ *P* < 0.05; ^**^ *P* < 0.01; ^***^ *P* < 0.001 by one-way ANOVA and LSD multiple comparison when comparing with baseline at the different weeks within each group. ^†^ *P* < 0.05; ^‡^ *P* < 0.01; ^§^ *P* < 0.001 by one-way ANOVA with significant difference between groups at each time point.

**Table 3:** HRV ANOVA results for age and time effects and the interaction of both effects for exercise group.

Parameters	Baseline		4th week		8th week		12th week		Interaction
	YG (*n* = 6)	MG (*n* = 14)	YG (*n* = 6)	MG (*n* = 14)	YG (*n* = 6)	MG (*n* = 14)	YG (*n* = 6)	MG (*n* = 14)	
TP (ms^2^)	10.7 ± 1.1	10.2 ± 0.9	10.9 ± 1.1	10.64 ± 0.8^*^	10.9 ± 0.8	10.54 ± 0.8	11.1 ± 1.0	11.22 ± 0.8^***^	0.295
LF (ms^2^)	10.0 ± 1.3	9.5 ± 1.3	10.2 ± 1.2	9.7 ± 1.1	10.2 ± 1.0	9.8 ± 1.0	10.5 ± 1.1	10.5 ± 1.0^***^	0.469
HF (ms^2^)	9.94 ± 1.2	9.3 ± 0.9^†^	10.13 ± 1.1	10.0 ± 0.9^***^	10.03 ± 0.9	9.7 ± 0.9^*^	10.2 ± 1.0	10.3 ± 0.9^***^	0.202
RRI (ms)	680.3 ± 192.9	730.4 ± 198.9	798.3 ± 117.1^*^	766.2 ± 186.5	769.3 ± 135.5	795.1 ± 187.2	809.8 ± 162.5^*^	812.1 ± 132.4^*^	0.665
SDNN (ms)	43.8 ± 20.6	38.6 ± 17.6	48.7 ± 28.2	48.5 ± 26.1	50.3 ± 29.9	42.8 ± 21.8	59.4 ± 28.9	59.2 ± 31.2^***^	0.852
RMSSD (ms)	40.6 ± 19.9	29.2 ± 13.0^†^	40.7 ± 18.7	43.7 ± 23.4^***^	41.1 ± 23.2	34.4 ± 15.74	43.1 ± 21.7	47.9 ± 23.4^***^	0.130
SDSD (ms)	41.0 ± 20.2	29.6 ± 13.1^†^	41.2 ± 19.0	44.2 ± 23.6^***^	41.6 ± 23.5	34.8 ± 15.91	43.6 ± 22.1	48.5 ± 23.71^***^	0.132
Weight (kg)	53.25 ± 10.7	55.26 ± 5.6	53.27 ± 10.1	55.11 ± 5.6	53.65 ± 10.5	55.48 ± 5.6	53.47 ± 9.8	55.44 ± 5.6	1.000
Body Fat (%)	27.68 ± 5.1	26.99 ± 6.6	26.67 ± 5.7	26.46 ± 6.5	26.73 ± 5.6	27.39 ± 6.0	27.37 ± 6.3	27.29 ± 6.4	0.992

Mean ± SD; YG: young group; MG: middle age group; TP: total power; LF: low-frequency power; HF: high-frequency power; RRI: R-R interval; SDNN: standard deviation of the normal-normal interval; RMSSD: square root of the mean of the sum of the squares of differences between adjacent normal-normal intervals; SDSD: standard deviation of differences between adjacent normal-normal intervals; ^*^ *P* < 0.05; ^***^ *P* < 0.001 by one-way ANOVA and LSD multiple comparison when comparing with baseline at the different weeks within each group. ^†^ *P* < 0.05; by one-way ANOVA with significant difference between groups at each time point.
